# Accuracy of Three Types of Apex Locators versus Digital Periapical Radiography for Working Length Determination in Maxillary Premolars: An In Vitro Study

**DOI:** 10.3390/clinpract12060107

**Published:** 2022-12-12

**Authors:** Masoumeh Ramezani, Marjan Bolbolian, Mohaddeseh Aliakbari, Ahad Alizadeh, Maryam Tofangchiha, Seyed Mohammad Faegh, Romeo Patini, Giuseppe D’Amato

**Affiliations:** 1 Department of Endodontics, Dental Caries Prevention Research Center, Qazvin University of Medical Sciences, Qazvin 34199-15315, Iran; 2 Student Research Committee, Qazvin University of Medical Sciences, Qazvin 34199-15315, Iran; 3 Medical Microbiology Research Center, Qazvin University of Medical Sciences, Qazvin 34199-15315, Iran; 4 Department of Oral and Maxillofacial Radiology, Dental Caries Prevention Research Center, Qazvin University of Medical Sciences, Qazvin 34199-15315, Iran; 5 Faculty of Stomatology, China Medical University, Shenyang 110001, China; 6 Department of Head, Neck and Sense Organs, School of Dentistry, Catholic University of Sacred Heart, 00135 Rome, Italy; 7 Unicamillus, International University of Health and Medical Sciences, 00131 Rome, Italy

**Keywords:** apex locator, digital radiography, working length

## Abstract

This study aimed to compare the accuracy of three types of apex locators versus digital radiography for working length (WL) determination. This experimental study was conducted on 58 extracted maxillary premolars. The teeth were decoronated, the access cavity was prepared, and WL was determined using a #15 K-file to serve as reference. The WL was then measured by Woodpex V, Woodpex III, and Root ZX apex locators in the presence of 0.9% saline, and also on a photostimulable phosphor plate (PSP) digital radiograph taken by the parallel technique. The values were compared with the actual WL using the paired *t*-test (alpha = 0.05). Digital radiography, Root ZX, Woodpex V, and Woodpex III determined the WL within ±0.5 mm from the actual value in 84.48%, 100%, 89.66%, and 87.93% of the cases, respectively. Woodpex V (*p* = 0.039), Woodpex III (*p* = 0.001), and Root ZX (*p* = 0.001) significantly over-estimated the WL. The WL measured on digital radiographs was not significantly different from the actual WL (*p* = 0.213). The position of the apical foramen (central/lateral) had no significant effect on the accuracy of WL determination by different techniques (*p* >0.05). Within the limitations of this in vitro study, all the tested modalities showed acceptable accuracy for WL determination in maxillary premolars.

## 1. Introduction

Precise cleaning of the root canal system and elimination of colonized microorganisms is the first step and a major goal in root canal therapy [1,2]. However, sterilization of the root canal system is not possible due to limitations of root canal disinfection techniques, instruments, and irrigants. Thus, cleaning of the root canal system is performed with the aim to decrease the intracanal microbial load in order not to interfere with periapical tissue healing [2,3]. Incorrect determination of working length (WL) can lead to complications such as postoperative pain and discomfort, the need for retreatment, and even tooth extraction. Thus, correct determination of WL is an imperative prerequisite for a successful endodontic treatment [4]. 

Several techniques and instruments are available for WL determination, such as the use of tactile sense, radiography, and apex locators [4]. Radiography is currently the most commonly used modality for WL determination in endodontic treatment. However, the main drawback of intraoral radiography is that it displays a two-dimensional image of a three-dimensional object and cannot precisely determine the exact location of the apex in teeth with lateral apical foramen [5]. As a result, the position of a file exceeding the actual WL of the canal may not be accurately detected on a radiograph due to superimposition and may lead to over-instrumentation and subsequent complications [6]. 

The apex locator is another commonly used instrument for WL determination. The recent advances in apex locators have enabled more accurate and predictable estimation of WL [7]. Apex locators can find the location of apical constriction, which is the most suitable location for termination of root canal instrumentation [7]. A meta-analysis evaluated four generations of apex locators and reported that all generations were accurate enough for measurement of WL, and generation of apex locators had no significant effect on their accuracy [8]. The root ZX apex locator has been the topic of numerous investigations and is currently the gold standard due to its exceptional accuracy [9,10]. However, it is costly and is not an option for many dental clinicians. Considering the significance of accurate WL determination in a successful endodontic treatment, this study aimed to assess the accuracy of Woodpex III and Woodpex V apex locators (low-cost possible alternatives) in comparison with Root ZX and digital photostimulable phosphor (PSP) radiography for determination of WL in maxillary premolars.

## 2. Methods 

This in vitro experimental study was conducted on 58 single-rooted maxillary premolars with one single canal or two canals (Vertucci’s type II with one apical foramen) that had been extracted as part of orthodontic treatment or due to poor periodontal prognosis. The sample size was calculated to be 58 according to a study by Kayabasi and Oznurhan [11], assuming alpha = 0.05 and a study power of 80% using PASS software 15 according to the method described by Nam and Blackwelder [12]. The inclusion criteria were single-rooted maxillary premolars with one single canal or two canals (Vertucci’s type II with one apical foramen) and no root caries, a mature apex, no root cracks, no resorption defects, and no history of previous endodontic treatment. Teeth with a root canal curvature > 30 degrees (measured according to Schneider’s method [13]), teeth with sclerotic or calcified canals, those without apical patency, and teeth with broken instruments in the canal were excluded. 

After collection, the teeth were immersed in 10% formalin, and debris and calculus were removed by an ultrasonic scaler. They were then immersed in 5.25% NaOCl for 2 h and subsequently stored in chloramine T solution [11,14]. The teeth were decoronated at the cementoenamel junction by a diamond disc to create a fixed and reproducible reference point for the measurements, and were coded. It should be mentioned that all the procedures and measurements were performed by a dental student under the supervision of an endodontist using a digital caliper (TNC, Hunan Co., Changsha, China) with 0.01 cm accuracy. A primary radiograph was obtained from the teeth by a dental X-ray unit (Planmeca, Helsinki, Finland) and DIGORA Optime DXR-60-01 PSP sensor (DIGORAOptime, Soredex, Finland) using the parallel technique such that the tip of the tube had an 8 cm distance from the tooth in the vertical position. The images were then observed using Scanora software, and the degree of canal curvature was calculated according to Schneider’s method [13]. Teeth with >30 degree root canal curvature were excluded. The absence of root canal calcification and presence of only one apical foramen were ensured on the radiographs. 

**Figure 1:**
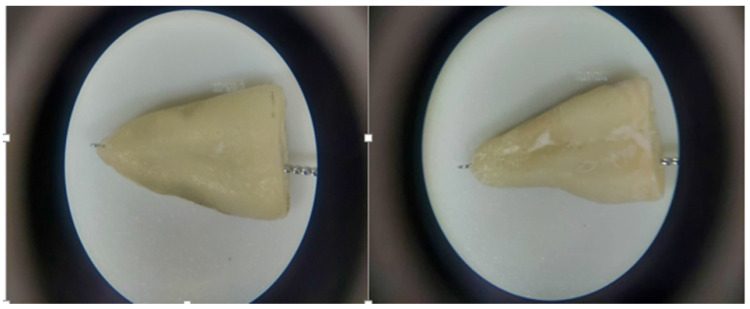
(**Left**) A tooth with lateral apical foramen; (**Right**) A tooth with central apical foramen.

The roots were then inspected under a stereomicroscope (LZOS, Lytkarino, Russia) at x6 magnification to ensure the patency of the apical foramen and determine its location [11]. Next, the teeth were assigned to two groups: 29 maxillary premolars with central apical foramen, and 29 maxillary premolars with lateral apical foramen (Figure 1). 

**Figure 2:**
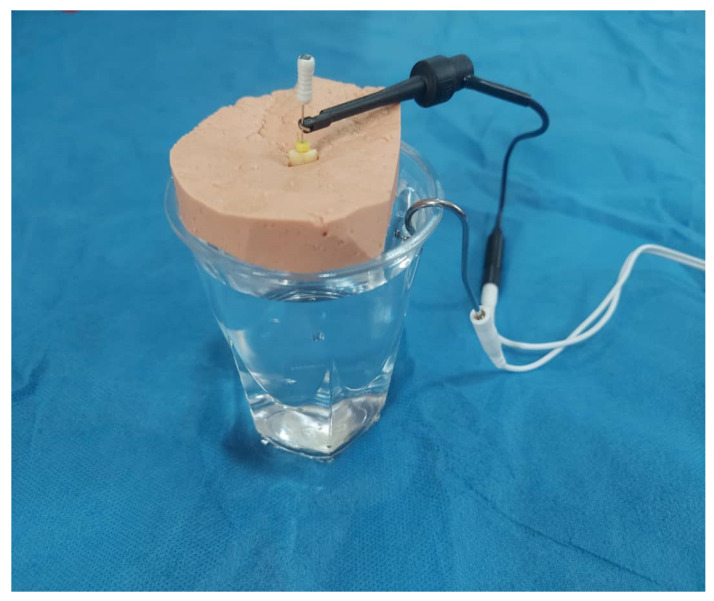
Experimental setup to measure the WL by the three apex locators.

In teeth with Vertucci’s type II root canals, one canal was considered as the reference for WL determination for higher accuracy. To measure the actual WL of the teeth (to serve as reference) and also for patency, a #15 K-file (Mani, Utsunomiya, Japan) was introduced into the canal until its tip was visible at the apical foramen under the stereomicroscope. The rubber stop was then adjusted at the cementoenamel junction, the file was removed, and its length was measured by a digital caliper. This technique was used as the gold standard for determination of actual WL. All measurements were repeated twice, and the mean value was recorded. Calcified teeth and those without patency were excluded and replaced. Next, the WL of each root canal was determined by Woodpex III (Woodpecker, Guilin, China), Woodpex V (Woodpecker, China), and Root ZX (Dentaport, J Morita, Kyoto, Japan) apex locators. For this purpose, the teeth were mounted in alginate molds to the level of their cementoenamel junction. To establish the current, the mounted roots were placed in containers filled with 0.9% saline such that the middle and apical parts of the roots were immersed in saline (Figure 2). Saline served as a conductor. The labial clip of the apex locator was also placed in saline [11,14]. All measurements were made by a #15 K-file twice, and the mean value was calculated. 

Next, the primary radiographs were used to measure the distance between the reference point and radiographic apex using Scanora version 5.0.2 software (Digora, Helsinki, Finland); 0.5 mm was subtracted from this length to determine the WL [11]. Another periapical radiograph was obtained from the teeth after introducing a #15 K-file into the canal. The distance between the file tip and radiographic apex was measured using the software ruler feature. If this distance was larger than 0.5 mm, the file position would be corrected, and radiography would be repeated until reaching the proper file length. Then, the file was removed from the canal, and its length was measured. The measurements were repeated twice, and the mean value was recorded. 

Statistical Analysis: Data were reported as mean and standard deviation for quantitative variables, and absolute and relative frequency values for qualitative variables. Since the normal distribution of WL data was ensured by the Shapiro-Wilk test, the accuracy of the five techniques in measurement of WL was compared by the repeated measures ANOVA and paired *t*-test. All statistical analyses were carried out using R software version 4.0.4. at the 0.05 level of significance.

## 3. Results

The mean actual WL (gold standard) was 12.24 ± 1.81 mm. The mean WL was 12.32 ± 1.83 mm as measured by Woodpex V, 12.49 ± 1.84 mm as measured by Woodpex III, 12.38 ± 1.84 mm as measured by Root ZX, and 12.29 ± 1.83 mm as measured on digital radiographs.

The maximum mean WL was reported by Woodpex III, and the minimum mean WL was measured on digital radiographs. Table 1 presents the frequency of differences > ±0.5 mm and >±1 mm between the measured values and the actual WL. Accordingly, the accuracy of Woodpex III, Woodpex V, Root ZX, and digital radiography was 87.93%, 89.66%, 100%, and 84.48%, respectively, for WL measurement within ±0.5 mm from the apical constriction. These values were 100%, 100%, 100%, and 96.55%, respectively, for WL measurement within ±1 mm from the apical constriction. All the measured values by the three apex locators were within ±1 mm distance from the apical constriction (100% agreement).

**Figure 3:**
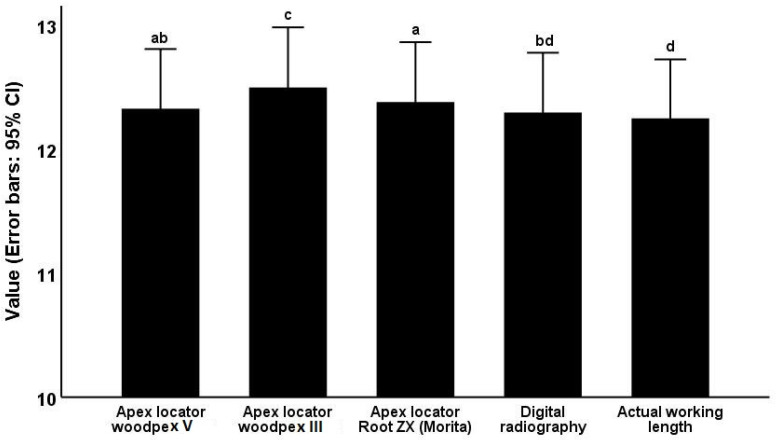
Comparison of the mean working length measured by the five modalities. Different letters for each pair of the groups represent a significant difference between them (*p* < 0.05).

Table 2 presents pairwise comparisons of the accuracy of five modalities regarding WL measurement. As shown, Woodpex V (*p* = 0.039), Woodpex III (*p* = 0.001), and Root ZX (*p* = 0.001) significantly over-estimated the WL. The WL measured on digital radiographs was not significantly different from the actual WL (*p* = 0.213). Additionally, the mean WL measured by Woodpex III was significantly greater than that measured by Root ZX (*p* = 0.002). The mean WL measured by Root ZX was also significantly greater than the value measured on digital radiographs (*p* = 0.026). The mean WL measured by Woodpex III was significantly greater than the value measured on digital radiographs (*p* = 0.001). The mean WL measured by Woodpex V was significantly lower than that measured by Woodpex III (*p* = 0.001) (Figure 3).

Table 3 presents the effect of position of the apical foramen (central/lateral) on the accuracy of WL measurement by different instruments. The position of the apical foramen had no significant effect on the accuracy of WL measurement by different instruments (*p* > 0.05).

**Figure 4:**
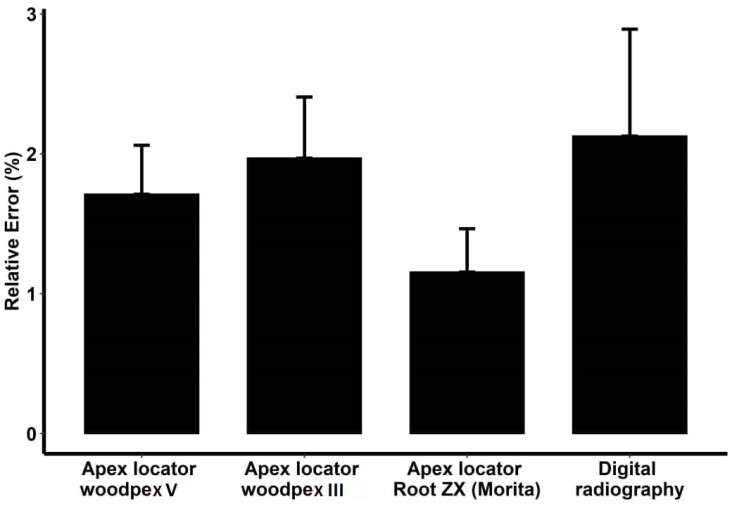
Relative error of the four modalities based on the actual working length in the samples with central apical foramen.

Comparison of relative error (deviation from the actual value) of the four measurement instruments compared with the actual value in teeth with the central apical foramen using the formula [abs (test − gold)/gold] × 100 revealed that the Root ZX apex locator was the most accurate for WL measurement in roots with the central apical foramen, with 1.155% error compared with the actual WL. Digital radiography had the lowest accuracy, with a 2.127% error rate compared with the actual WL (Figure 4).

**Figure 5:**
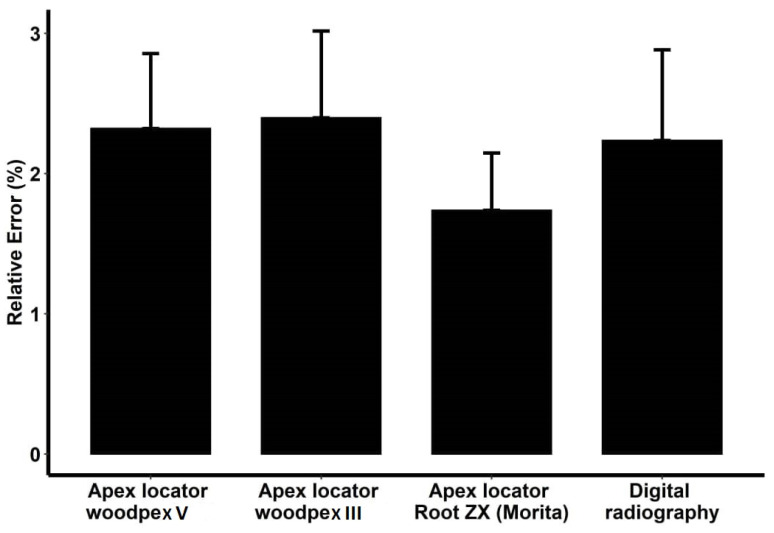
Relative error of the four modalities based on the actual working length in the samples with lateral apical foramen.

The most accurate tool for measurement of WL in teeth with the lateral apical foramen was the Root ZX apex locator, with a 1.739% error rate compared with the actual WL, while Woodpex III had the lowest accuracy, with a 2.398% error rate compared with the actual WL (Figure 5).

In total (irrespective of apical foramen position), Root ZX was found to be the most accurate tool for measurement of WL, with a 1.447% error rate, and Woodpex III was found to be the least accurate tool for measurement of WL, with a 2.184% error rate, followed by digital radiography, with a 2.182% error rate compared with the actual WL.

## 4. Discussion

This study assessed the accuracy of Woodpex III (third generation) and Woodpex V (fifth generation) apex locators in comparison with Root ZX (third generation) and digital PSP radiography for determination of WL in maxillary premolars.

As mentioned earlier, Nasiri et al. [8], in their meta-analysis, showed no significant effect of the generation of apex locators on their accuracy. Thus, in the present study, the generation of apex locators could not serve as a confounding factor. The results showed that all modalities overestimated the WL. Pairwise comparisons showed significant differences between the actual WL and the values measured by Woodpex V, Woodpex III, and Root ZX.

A search of the literature by the authors yielded no study on the accuracy of Woodpex V and Woodpex III apex locators. Nahidi et al. [15] only mentioned using a Woodpecker apex locator and did not disclose its model/generation. In addition, Suprastiwi and Meidyawati [16] used Woodpex I, which was different from the apex locators used in the present study. Thus, the present results could not be compared with their findings. In the present study, Root ZX significantly overestimated the WL (*p* = 0.001). Cianconi et al. [17] and Guise et al. [18] found a significant difference between the WL measured by Root ZX and the actual WL. Cianconi et al. [17] evaluated 101 extracted teeth of different types and reported that Root ZX overestimated the WL, which was in line with the present findings. Nahidi et al. [15] found no significant difference in the WL measured by Root ZX and the actual WL in single canal maxillary incisors. Although it has been reported that Root ZX overestimates the WL, it should be mentioned that the margin of error within 1 mm is clinically acceptable because evidence shows that apical constriction is located within this range [17,19,20,21]. Difference in the results of the two studies may be due to differences in sample size, type of tooth, and method of measurement of the actual WL (use of histological sections in the study by Nahidi et al. [15]). Mahmoud et al. [22] evaluated 35 mandibular first premolar teeth and mounted them in a mold to simulate the intraoral environment. They found no significant difference in the mean WL measured by Root ZX and the actual WL, which was different from the present findings probably due to differences in tooth type, sample size, and medium used for the measurement of WL by the apex locator.

The present results indicated that digital radiography overestimated the mean WL but it was not statistically significant. Yadav et al. [1] indicated that both digital and conventional radiography overestimated the WL. Similarly, Faraj [5], Elshinawy [23], and Mahmoud et al. [22] showed that the conventional and digital radiography overestimated the WL. It should be noted that the majority of the available studies on this topic reported the mean WL of the canals [1,22,24], which is not highly accurate because the presence of a large difference between the measured and the actual WL in even one tooth can significantly affect the calculated mean value. To overcome this problem, in the present study the relative error (error rate relative to the actual WL) was calculated in addition to the mean difference to more precisely assess the accuracy of the methods. To calculate the relative error, each individually measured WL was subtracted from the actual WL, and the percentage of error compared with the actual WL of the canals was calculated using the respective formula. The results showed that among the four modalities, digital radiography yielded values closer to the actual WL, while according to the calculated percentage of relative error, the Root ZX apex locator had the maximum accuracy (1.447%) among the tested modalities. This finding was in agreement with the results of Yilmaz et al. [25] and Elshinawy et al. [23]. The minimum accuracy belonged to the Woodpex III apex locator (2.184%) and digital radiography (2.182%).

A ±0.5 mm difference between the measured and the actual WL is acceptable in endodontic treatment [26]. According to the present results, digital radiography, the Root ZX apex locator, the Woodpex V apex locator, and the Woodpex III apex locator determined the WL within a ±0.5 mm difference from the actual value in 84.48%, 100%, 89.66%, and 87.93% of the cases, respectively. All measurements made by the three apex locators had a ±1 mm difference from the actual value. This finding was in agreement with the results of previous studies reporting 73% to 98% accuracy for apex locators for WL determination within a ±1 mm distance from the apical constriction [27,28]. In the present study, the accuracy of Root ZX for WL determination within a 0.5 and 1 mm distance from the apical constriction was 100%; this value was 96.2% in the study by Shabahang et al. [19]. Mahmoud et al. [22] reported the accuracy of Root ZX to be 71.43% for WL determination within a 0.5 mm distance from the apical foramen. The difference between the value reported in their study and the value obtained in the present study may be due to different environments where the measurements were made, or differences in sample size, applied statistical methods, and type of teeth. The accuracy of Root ZX was 76.7% and 100% for WL determination within a 0.5 and 1 mm distance from the apex, respectively, in the study by Nahidi et al. [15], which was different from the value reported in the present study due to different types of teeth, sample size, and method of measurement of the actual WL. The position of the apical foramen, canal obstruction, diameter of apical foramen, pre-flaring of the coronal third, dry/moist root canal environment, and size of file are among other factors that can cause variations in the accuracy of apex locators reported in the literature [29].

The effect of the position of the apical foramen (central and lateral) on the accuracy of WL determination by different modalities was also evaluated in the present study. The lateral position is the most common position of the apical foramen in maxillary first and second premolars. The results showed that the mean measured WL in teeth with the lateral apical foramen was greater than that in teeth with the central apical foramen for all modalities; however, no significant correlation existed between the position of the apical foramen and the accuracy of WL determination. ElAyouti et al. [30] reported that radiography overestimated the WL when the position of the anatomic apex and apical foramen did not match. This more commonly occurs in teeth with the lateral apical foramen. Similarly, Pagavino et al. [31] reported that Root ZX overestimated the WL in teeth with the lateral apical foramen. The effect of the position of the apical foramen relative to the original axis of the root on the accuracy of apex locators has been previously confirmed [31,32]. It has also been reported that the diameter of the apical foramen and its lateral position may negatively affect the accuracy of apex locators [32].

The present study showed that the percentage of error in all modalities was higher in teeth with the lateral apical foramen, which was in agreement with the results of limited studies available on this topic [31,32]. Pagavino et al. [31] reported significantly higher accuracy of Root ZX in teeth with the central apical foramen compared with those with the lateral apical foramen. Piasecki et al. [32] indicated a higher percentage of error of Root ZX and Apex Id apex locators in teeth with the lateral apical foramen; however, the position of the apical foramen had no statistically significant effect on the accuracy of apex locators. In the present study, Root ZX had maximum and digital radiography had minimum accuracy in WL determination in teeth with the central apical foramen while Root ZX and Woodpex III had the maximum and minimum accuracy, respectively, in determination of WL in teeth with the lateral apical foramen.

This study had several strengths. According to Wrbas et al. [33], comparison of the accuracy of different apex locators in the determination of WL is only feasible if similar teeth are evaluated. Thus, this study evaluated single-rooted maxillary premolars with a root curvature < 30 degrees. Also, evidence shows that changing the file diameter affects the accuracy of WL determination [34]. Thus, all measurements were made by a #15 K-file in the present study. Furthermore, the testing conditions were the same for all teeth to minimize errors in measurements.

Since this study had an in vitro design, the confounding effect of difficulty in taking the radiographs in the clinical setting and the superimposition of anatomical structures such as the zygomatic arch and the maxillary sinus on the roots could not be assessed. Therefore, generalization of the results to the clinical setting must be done with caution.

As mentioned earlier, no previous study has assessed the accuracy of Woodpex III and V, thus disallowing comparison of our results. Thus, further studies are required on this topic. Moreover, similar in vivo studies on teeth scheduled for extraction are required to obtain more reliable results. The accuracy of apex locators based on the size of the file, the correlation of the accuracy of apex locators and canal curvature, the accuracy of apex locators in teeth with periapical lesions or open apices, the effect of additional canals and their number on the accuracy of apex locators, and the effect of the presence of blood, chlorhexidine, or other irrigants instead of saline in the canal on the accuracy of apex locators are all interesting topics for further research in this field.

## 5. Conclusions

Within the limitations of this in vitro study, all the tested modalities showed acceptable accuracy for WL determination in maxillary premolars. Root ZX was the most accurate modality for WL determination. However, considering the comparably high accuracy of Woodpex V, its lower cost than Root ZX, and easier availability in dental markets, it may be used as an acceptable adjunct to radiography. Moreover, in the case of the presence of a discrepancy in WL as measured by Root ZX and Woodpex V and digital radiography in maxillary premolars, the value displayed by these apex locators is likely more reliable than the value measured on digital radiographs.

## Figures and Tables

**Table 1:** Frequency of differences > ±0.5 mm and > ±1 mm between the measured values and the actual working length (WL*).

Instrument	Difference > ±0.5 mm and ˂±1 from the Actual WL*		Difference ≥ ±1 mm from the Actual WL*	
	Frequency	Percentage	Frequency	Percentage
Woodpex V	6	10.34	0	0
Woodpex III	7	12.07	0	0
Root ZX	0	0	0	0
Digital radiography	9	15.52	2	3.45

**Table 2:** Pairwise comparisons of the accuracy of five modalities regarding working length measurement.

Comparison	Difference (Effect Size)	Standard Deviation	Degree of Freedom	T	*p*-Value
Woodpex V—Actual	0.07810345	0.037658	228	2.074	0.039
Woodpex III—Actual	0.24931034	0.037658	228	6.6204	<0.001
Root ZX—Actual	0.13172414	0.037658	228	3.4979	0.001
Digital radiography—Actual	0.04706897	0.037658	228	1.2499	0.213
Woodpex V—Root ZX	−0.0536207	0.037658	228	−1.424	0.156
Woodpex III—Root ZX	0.11758621	0.037658	228	3.1225	0.002
Digital radiography—Root ZX	−0.0846551	0.037658	228	2.248	0.026
Woodpex V—Digital radiography	0.03103448	0.037658	228	0.8241	0.411
Woodpex III—Digital radiography	0.20224138	0.037658	228	5.3705	<0.001
Woodpex V—Woodpex III	−0.1712069	0.037658	228	−4.546	<0.001

**Table 3:** Effect of position of apical foramen (central/lateral) on the accuracy of working length measurement by different instruments.

Position		Central (Mean and Std. Deviation)	Lateral (Mean and Std. Deviation)	*p*-Value
	Instrument			
Woodpex V		12.211 ± 1.814	12.443 ± 1.875	0.634
Woodpex III		12.417 ± 1.827	12.580 ± 1.894	0.740
Root ZX		12.284 ± 1.819	12.478 ± 1.888	0.693
Digital radiography		12.134 ± 1.791	12.459 ± 1.899	0.506
Actual		12.179 ± 1.765	12.319 ± 1.883	0.771

## Data Availability

The data used to support the findings of this study are included within the article.
